# Competition between a Lawn-Forming *Cynodon dactylon* and a Tufted Grass Species *Hyparrhenia hirta* on a South-African Dystrophic Savanna

**DOI:** 10.1371/journal.pone.0140789

**Published:** 2015-10-28

**Authors:** J. A. Zwerts, H. H. T. Prins, D. Bomhoff, I. Verhagen, J. M. Swart, W. F. de Boer

**Affiliations:** 1 Resource Ecology Group, Wageningen University, Droevendaalsesteeg 3a, 6708PB, Wageningen, the Netherlands; 2 School of Life Sciences, University of Kwazulu-Natal, Westville Campus, Private Bag X54001, Durban, 4000, South Africa; 3 Welgevonden Game Reserve, PO Box 433, Vaalwater, 0530, South Africa; Helmholtz Centre for Environmental Research - UFZ, GERMANY

## Abstract

South African savanna grasslands are often characterised by indigestible tufted grass species whereas lawn grasses are far more desirable in terms of herbivore sustenance. We aimed to investigate the role of nutrients and/or the disturbance (grazing, trampling) by herbivores on the formation of grazing lawns. We conducted a series of common garden experiments to test the effect of nutrients on interspecific competition between a typical lawn-forming grass species (*Cynodon dactylon*) and a species that is frequently found outside grazing lawns (*Hyparrhenia hirta*), and tested for the effect of herbivore disturbance in the form of trampling and clipping. We also performed a vegetation and herbivore survey to apply experimentally derived insights to field observations. Our results showed that interspecific competition was not affected by soil nutrient concentrations. *C*. *dactylon* did show much more resilience to disturbance than *H*. *hirta*, presumably due to the regenerative capacity of its rhizomes. Results from the field survey were in line with these findings, describing a correlation between herbivore pressure and *C*. *dactylon* abundance. We conclude that herbivore disturbance, and not soil nutrients, provide *C*. *dactylon* with a competitive advantage over *H*. *hirta*, due to vegetative regeneration from its rhizomes. This provides evidence for the importance of concentrated, high herbivore densities for the creation and maintenance of grazing lawns.

## Introduction

Grazing lawns consist of grass species which by means of continuous cropping remain in a highly productive state, resulting in the availability of nutritious, low fibre regrowth [1,2]. Increasing the abundance of these productive and nutritious species may increase the intake rate of herbivores [3]. Grazing lawns are an important feature of the landscape both in the temperate zone [4,5] and in tropical savannas [6–8]. The lawn-forming grasses, as for example *Cynodon dactylon*, generally have a low stem content and dense biomass (i.e., much mass per volume of sward [9]) and are preferred over other grass species by selective grazers [10,11]. Contrasting are the tufted grasses, as for instance *Hyparrhenia hirta*. These perennial grasses, are less desirable due to their high stem content that lowers forage intake for grazers and consequently limits the nutrient intake that is derived per unit effort [3,9].

Although grazing lawns are important for herbivores and for savanna functioning [12] it is not very clear how lawn-forming grasses are able to outcompete tufted grasses at specific sites. Soil fertility has been proposed as an important ecological factor in shaping grass assemblages [13], but it is still unknown if competition for soil nutrients alone can lead to transitions from tall grass communities to short grass communities or vice versa [13,14]. When nutrients are limiting, a species can outcompete other species in the uptake of N or P, resulting in asymmetric resource competition by monopolizing these nutrients for plant growth, maintenance or seed production [15,16]. This species will then become dominant as it harvests more light and outgrows its competitors [17]. So, lawn-forming grasses might be out-shaded by tufted grasses due to their larger stature, if grazing does not limit the growth of these longer tufted grasses [13,14]. Raising nutrient levels, or changing grazing or disturbance levels is expected to shift species dominance towards these lawn-forming grasses. It has been shown that grazing lawns can be established by nitrogen (N) and phosphorus (P) fertilization [12,18], thereby releasing low competitive species from competition. However, when grazers are excluded from plots with additional nutrients, tufted grasses again became dominant [19,20,21]. Lawn grasses show compensatory growth in response to grazing in contrast with the grazing-sensitive tufted grasses [12]. Hence, additional nutrients in combination with grazing (or mowing) treatments might be able to trigger the desired changes, but only when a sufficiently high grazing pressure follows the fertilization [12,18,19].

Lawn and tufted grasses thus have different mechanisms to cope with herbivory. Tufted grasses are relatively unattractive for herbivores (due to their high stem content), whereas lawn grasses have developed mechanisms to facilitate persistence and regrowth under constant defoliation [22,23]. The latter has the ability to form rhizomes (belowground) and stolons (aboveground) which are lateral shoots that can be formed from the same axillary bud that also forms orthotropic shoots. These plant parts have been ascribed storage [24,25] and forage functions [26,27], enable the species to colonize new patches [21] and have furthermore been associated with resilience to disturbance [11,24]. Resilience is realised through the regenerative capacity of these plant parts, which contribute to regrowth under heavy disturbance. However, it has been shown that when trampled on, stolons suffer just as much damage as other aboveground biomass and then subsequently take one whole year to grow back to their previous state [28]. They are therefore not considered to play a large role in resilience to disturbance by herbivores. Underground plant parts like rhizomes are less affected by grazers and are more likely to play an important part in plant survival under heavy grazing [21].

Contrary results pointing to the isolated importance of different driving mechanisms for lawn formation, i.e. nutrients, herbivory and competition remain unclear about the respective strengths and relations of these drivers to one another [13,14,21,22]. This study aims to provide clarity on the importance of each of these drivers for lawn formation by studying them both combined and in isolation of each other, as well as by having a closer look at plant specific features such as stolons and rhizomes and the role they play herein. To investigate what the competitive advantages are of a lawn-forming grass species compared to a tufted grass species, we conducted common garden experiments and a field study to examine the effects of nutrients and herbivore impact on the interspecific competition between *C*. *dactylon* (a lawn grass species) and *H*. *hirta* (a tufted grass species), two widely distributed African grass species [29]. We hypothesized that under a combination of increased herbivore impact in the form of trampling and grazing, and with increasing Nitrogen (N) and/or Phosphorus (P) fertilization, the cover of the lawn grass *C*. *dactylon* in a sward increases to the detriment of the tufted grass H. *hirta*.

## Material and Methods

The study was conducted in the Welgevonden private game reserve (24°10’S; 27°45’E to 24°25’S; 27°56’E), South Africa with a subtropical climate and a mean annual rainfall of 620 mm. The area has been subject to grazing lawn related research for a number of years, of which the original 2007 research design consisted of 45 plots of 100mx100m of nitrogen, phosphorus or no fertilization in combination with mowing treatments once, twice or three times a year. Each combination of treatments has 5 replicates, that are spread out over the reserve.

We performed a common garden experiment in March—June 2012. The soil used was gathered from nearby each replicate and was then crushed, mixed, and sieved to homogenize the soil for the experimental set-up. Similar-sized individual grass plants (*C*. *dactylon* and *H*. *hirta*) were gathered in the field and were then planted in 9 l soil bags (35cm high, soil surface: 260cm^2^). After transplanting, the plants had a 14-day period to get well-established before the experimental treatments started. During the establishment period, a shade net covered the common garden plot and all plants were cut down to 5 cm in height to minimize heat stress and dehydration. Watering was done manually on a daily basis to ensure that soils were kept constantly at field capacity, which was assessed visually and manually. The plot was inaccessible to herbivores and termite damage was prevented by regular relocation of the soil bags.

For all the experiments it was noted when a plant died, which was defined as the absence of any green aboveground grass tissue. After a period of seven weeks, the treatments of experiment 1 and 2 were terminated and the grasses were harvested and cleared of sand by submerging them in water. All rhizomes were harvested after the experiment and inspected for shoot development. Plants were separated in above and belowground plant parts which were dried at 70°C for 48 h after which the dry weight was measured. The role of a difference in seed banks between the two species was not investigated in this study.

### Interactions and feedback of mowing, fertilization and herbivory

#### Competition under N and P Fertilization

Experiment 1 had a 2-factor fully randomized design consisting of 4 fertilization treatments and a control (Nitrogen: N, and Phosphorus: P; both treatments either intense: ++, or less intense: +; Table 1), using commercially available fertilizer. Each bag contained 6 individual plants that were planted in a circle; either as monoculture swards (*C*. *dactylon* or *H*. *hirta*) or as mixed swards with alternating species. Each combination of a fertilization treatment and species composition, was replicated 10 times, totalling 150 bags. In order to improve nutrient availability for plant uptake, the soil pH (originally pH 3.8–4.3) was increased with 6.6g lime (Allgro Agricultural Lime, Ca 176g/kg). Bags were moved randomly each day over the experimental area, to avoid site effects. Per bag, one averaged sized individual was selected per species to measure the number of leaves, length of longest leaf and the number of culms every 2^nd^ week. After the experiment was terminated, the aboveground biomass of all plants was clipped, dried and weighed, and available N and P content in the soil was measured (S1 Table).

**Table 1:** Nutrient dosages of the fertilization treatments and lime addition. One bag is 9 liter of soil. Used fertilizer: LAN (Limestone ammonium nitrate; 28% nitrogen); and superphosphate (10.5% phosphate).

Treatments	Dosage per bag (g/bag)	Dosage per hectare (kg/ha)	Nutrient content fertilizer (g/kg)	Lime (g/bag)
N++	1.6	600	280	6.6
N+	0.66	250	280	6.6
P++	1.32	500	83	6.6
P+	0.33	125	83	6.6
Control	No fertilization	No fertilization	No fertilization	6.6

#### Herbivore Disturbance and Rhizome Function

To investigate the tolerance to herbivore impact of the two grass species and to assess whether rhizomes play a role herein, we carried out a second experiment, which had a 2-factor fully randomized design with the factor ‘reserve-organ’ consisting of three levels and the factor ‘treatment by simulated trampling and grazing’ with two levels; each unique treatment combination was replicated nine times. The levels of the factor reserve-organ were *C*. *dactylon* in its normal condition with rhizomes, *C*. *dactylon* from which the rhizomes were manually removed, and *H*. *hirta*. The treatment simulated trampling and grazing consisted of with or without trampling and clipping every 2^nd^ day, a highly intense treatment to mimic herbivore disturbance as a very stressful condition with clear effects on plant survival. Grazing was simulated by clipping [30], i.e., all aboveground material was clipped at a height of 5 cm, including the control at the initiation of the experiment to equalize the starting position of all plants. Trampling was simulated by repeatedly dropping a 10 kg wooden log, with a wildebeest hoof-shaped carving at the bottom, from a height of 50 cm. By imitating a herbivore’s stepping in a standardized way, this technique mimics the mechanical damage a herbivore imposes on the sward and the effect of soil compaction by the subsequent pressure of its body mass in a repeatable manner.

To investigate the function of rhizomes for *C*. *dactylon* an intact root system was required. Therefore whole sods of grass instead of single shoots were taken out of the field to a depth of 20 cm. *C*. *dactylon* plants are all interconnected in a sod via stolons and/or rhizomes, whereas the individual *H*. *hirta* plants are all independent tufts. For *C*. *dactylon* this meant that a whole sod of interconnected shoots with a diameter of 20 cm was transferred, whereas for *H*. *hirta* one similar sized tuft was used for the experiments.

This experiment had two different levels for the *C*. *dactylon* sods: one had its root system entirely intact, whereas the other had the majority of its connecting rhizomes removed while the rest of the sod structure remained intact. This last procedure was done by shaking the bulk part of the sand off the roots, by gently bouncing the entire sod on a flat surface and repeatedly removing the loose sand. After this, the majority of the rhizomes were cut off from below with a long pair of scissors. Even though only half of the *C*. *dactylon* sods were subject to this treatment, all sods were cleared of their soil prior to being replanted, in order to standardize all treatments.

Every 2^nd^ week the number of leaves and the number of resprouts were counted, but not the height and the length of the longest leaf because clipping treatments were applied so that these variables would not be good indicators of growth.

#### Rhizome Regeneration

The third experiment was a growth experiment to assess the regenerative capacity of rhizomes of *C*. *dactylon* that are disconnected from any aboveground material. This experiment was designed to give insight in to what extent rhizomes are dependent on remaining aboveground material for their survival and regeneration, thus measuring the resilience of *C*. *dactylon* (unmatched by tufted grasses because they do not have rhizomes). A hundred pieces of rhizome with a length of 20 cm and similar thickness (5–10 mm) were buried 10 cm underground and grown for a period of ten weeks. This was done in five separate 21 l soil bags with a soil surface of 450 cm^2^. Every 2^nd^ week the number of resprouts were counted.

Bags from all three experiments were placed randomly in the experimental area, and were moved frequently around to avoid site-effects.

#### Vegetation Cover

A vegetation survey in existing experimental plots with grazing lawns was carried out in combination with a survey of the herbivore dung density to study the effects of mowing and fertilization on herbivore visitation and grass composition. The vegetation survey was done from March until June 2012. For the survey, fifteen 100x100 m plots, which were distributed over 5 sites, all mown twice (mowed in December and February at about 5 cm grass height) and either fertilized with N, or P or not fertilized, were sampled. Nitrogen was applied with 250 kg N/ha using LAN (Limestone ammonium nitrate; 28% nitrogen). The phosphate was given as 125kg P/ha superphosphate (10.5% phosphate). Fertilization was done at the end of January. Next to these fifteen plots, five additional plots of 100mx100m (one per site) were set out in the matrix vegetation. These plots were not fertilized and not mown, set out on the same plains on locations at comparable elevation and slope but at a distance of 100 m from the nearest plot to minimize spill-over effects [31]. Each plot was walked through eight times at evenly spaced transects 12.5 m apart from one another. On each transect there were eight points of measurement, also evenly spaced and separated from one another by a distance of 12.5 m. This spatial layout allowed for distinguishing between edges and central areas since these areas are likely to be differentially affected by herbivores as animals might concentrate in central areas of mown plots (pers. obs.) where they apparently can maintain an overview of the surrounding areas so that chances of predator detection are increased [32]. A difference in herbivore pressure between edge and central areas is, however, only to be expected when the area that surrounds the plot is covered with high enough vegetation to obstruct the overview of herbivores. Therefore a distinction was made between plots with high (> 1m) surrounding vegetation and low (< 1 m) surrounding vegetation. Sampling was done by the point interception method [33,34], with a square frame of 0.5x0.5 m with 25 pins in a grid at equally spaced points, to record the species composition, or if no vegetation was present, bare soil.

#### Herbivore Pressure

Surveying the density of herbivore pressure was done using faecal sampling for Burchell’s Zebra (*Equus burchellii)* and Blue wildebeest *(Connochaetus taurinus*), the dominant grazers at Welgevonden. No distinction was made between the droppings of the separate species and herbivore density was expressed as droppings per sample point. The counts were performed at the same sampling points as for the vegetation survey, where the number of droppings in a circle with a 5 m radius was recorded. Recordings were done twice over a three week interval, using the accumulated dropping densities as dependent variable which partly corrects for day-to-day differences in visitation rates. Droppings were not removed between the recordings. This was compensated for by omitting old droppings during the second count after visually establishing the decay speed over a three week period beforehand.

#### Nutrients

Soil nutrient data were used from previous research conducted in Welgevonden (October 2011—January 2012), where anion exchange resin bags were used to assess the available N (NH_4_ and NO_3_) and P (PO_4_) content in the soil at 10cm depth in the experimental 100x100m plots. The resin bags consisted of 3g of resin which was placed in nylon bags. Four resin bags were placed in each 100x100m plot; one in each corner and 15m from the edge of the plot. In total 15 plots (5 N-fertilized, 5 P-fertilized, 5 non-fertilized) received resin bags, resulting in a total of 60 samples. The bags were retrieved after 8 weeks, washed to remove the soil and dried at 25°C. N and P mineralization was determined by an extraction method with potassium chloride (KCl) and analyzed in a colorimeter [35]. The soil nutrient data together with the cover data of the species *C*. *dactylon* and *H*. *hirta* and the bare soil was used to determine if there was a correlation between nutrient availability in the soil and cover.

### Statistics

Statistics were computed with IBM SPSS 20.0. Tests were always full factorial with a backward elimination of variables that were not significant (*p*>0.05).

#### Competition under N and P Fertilization

Number of leaves and culms, length of longest leaf and aboveground biomass were determined during the experiment to assess the effect of competition and nutrients on plant growth and morphology. The changes of these factors between the first and last measurements as well as the proportional changes of both leaves and culms (calculated by dividing final and initial quantities), were calculated, followed by a log-transformation to obtain a normal distribution. A full factorial ANCOVA model was used to test for the effect of ‘competition’, ‘fertilization’ and their interaction on the species performance, with the natural logarithm of the total dry weight per plant as covariate. As pots were moved randomly over the experimental area each day to avoid site effects, we did not include a random block effect in the analysis. A Sidak post-hoc test was used to distinguish the homogenous subsets.

#### Performance under Herbivore Disturbance

*H*. *hirta* was not considered in statistical tests due to high mortality rate. To test for the effect of herbivory, reserve organ and their interaction on the number of leaves for *C*. *dactylon*, the first measurement of the number of leaves was used as a reference point to which the subsequent measurements were related to show the proportional increase or decrease of the number of leaves. Over these proportional changes a two-way ANCOVA was carried out after a square root-transformation was conducted to attain a normal distribution, with ‘time’ as covariate to study the reaction time of these proportional changes. The number of resprouts followed a Poisson distribution, and a Poisson regression was used to test treatment effects on the number of resprouts. Differences in biomass after termination of the experiment were analysed with a two-way ANOVA for which the aboveground biomass required a square root-transformation and belowground biomass data required a ln-transformation to obtain normally distributed residuals.

#### Rhizome Regeneration

For the increase of aboveground shoots (*y*) over time (*t*) an inverse regression was used (*y* = *β*_0_+*β*_1_/*t*) to take the non-linearity of the data into account.

#### Vegetation Cover, Herbivore Pressure and Nutrients

A Generalized Linear Model (GLM) with a nested design was used to test for the effect of surrounding vegetation height, position within the plot, treatment (moved/not fertilized, mowed/P-fertilization, mowed/N-fertilization), and dung densities on *C*. *dactylon*, and *H*. *hirta*. A similar nested GLM was carried out to explain differences in dung densities. A Spearman rank correlation coefficient [36] was calculated to test for the correlation between mean percentage cover per species in the fifteen 100mx100m plots and the mean concentrations of the available N and P content in the soil in these fifteen plots, because data deviated from a normal distribution even after an arcsine-transformation.

## Results

### Competition under N and P Fertilization

No effect of competition or fertilization was found on the aboveground biomass of *H*. *hirta*, but there was as small competition x fertilization interaction effect on the biomass of *C*. *dactylon* (R^2^_adj_ = 0.177, *F*_*4*,*90*_ = 3.583, *p* = 0.009; Table 2), with a lower amount of biomass of *C*. *dactylon* when growing in a mixed sward under P fertilization.

**Table 2:** Mean (SE) aboveground biomass (g DW/plant) and proportional change in the number of leaves, length of the longest leaf and the number of culms as the result of the two competition treatments and the five fertilization treatments after 7 weeks (*n* = 10 bags, for each unique combination of treatments).

	*H*. *hirta*		*C*. *dactylon*	
Treatment	Mixed sward	Mono-culture	Mixed sward	Mono-culture
*Biomass*				
N++	5.98 (0.55)	6.27 (1.03)	0.81 (0.10)	0.64 (0.11)
N+	6.68 (1.29)	6.35 (0.91)	0.71 (0.09)	0.61 (0.05)
P++	6.92 (1.08)	5.96 (0.73)	0.40 (0.08)	0.69 (0.14)
P+	6.82 (1.17)	6.88 (1.18)	0.39 (0.06)	0.64 (0.06)
Control	4.77 (0.63)	5.68 (0.39)	0.48 (0.07)	0.57 (0.08)
*Number of leaves*				
N++	0.65 (0.08)	0.53 (0.07)	1.00 (0.14)	0.69 (0.16)
N+	0.74 (0.13)	0.55 (0.16)	1.14 (0.08)	0.95 (0.11)
P++	0.54 (0.13)	0.40 (0.08)	0.66 (0.21)	0.97 (0.14)
P+	0.52 (0.09)	0.59 (0.08)	0.59 (0.12)	1.05 (0.09)
Control	0.55 (0.08)	0.36 (0.09)	0.64 (0.11)	0.78 (0.13)
*Length longest leaf*				
N++	0.34 (0.06)	0.3 (0.06)	0.46 (0.13)	0.45 (0.06)
N+	0.34 (0.06)	0.22 (0.06)	0.70 (0.07)	0.47 (0.08)
P++	0.21 (0.08)	0.11 (0.05)	0.44 (0.10)	0.47 (0.07)
P+	0.22 (0.06)	0.33 (0.07)	0.32 (0.11)	0.63 (0.06)
Control	0.24 (0.06)	0.24 (0.05)	0.51 (0.12)	0.38 (0.08)
*Number of culms*				
N++	0.81 (0.18)	0.47 (0.11)	7.75 (1.96)	4.32 (0.53)
N+	0.51 (0.13)	0.40 (0.13)	10.16 (2.13)	6.67 (1.56)
P++	0.41 (0.17)	0.51 (0.13)	5.69 (1.99)	5.44 (1.22)
P+	0.46 (0.09)	0.20 (0.06)	2.75 (0.60)	7.57 (2.15)
Control	0.37 (0.11)	0.18 (0.06)	4.84 (0.97)	7.13 (2.09)

The response in the number of leaves differed between the species (Table 2, S2 Table). For *C*. *dactylon* under nitrogen treatment the number of leaves was lower in the monoculture whereas under phosphorus fertilization the number of leaves was higher in the monoculture (interaction; R^2^_adj_ = 0.098, *F*_*9*.*86*_ = 2.099, *p* = 0.038). *H*. *hirta* showed an increased number of leaves in the mixed sward regardless of the type of fertilization (R^2^_adj_ = 0.062, *F*_*1*,*82*_ = 4.372, *p* = 0.040).

For *C*. *dactylon*, the length of the longest leaf increased under low nitrogen fertilization in the mixed sward (interaction; R^2^_adj_ = 0.063, *F*_*4*,*86*_ = 2.747, *p* = 0.033). For the length of the longest leaf of *H*. *hirta*, no significant model was found.

For *C*. *dactylon*, the number of culms was higher in a mixed sward under nitrogen fertilization, as well as for the monoculture under low phosphorus fertilization (interaction; R^2^_adj_ = 0.104, *F*_*9*,*86*_ = 2.340, *p* = 0.021). For the number of culms of *H*. *hirta*, no significant model was found.

#### Performance under Herbivore Disturbance

Clipping and trampling had a negative effect on survival and the number of leaves of both species. *C*. *dactylon* with rhizomes was affected less than when rhizomes were missing and *H*. *hirta* was affected the most: all replicates of both *C*. *dactylon* groups survived whereas for *H*. *hirta* nearly all replicates died by the end of the experiment, with serious declines in survival rates starting after 6 weeks (Table 3).

**Table 3:** Survival percentages for the clipped and artificially trampled *C*. *dactylon* with and without rhizomes and *H*. *hirta* over time (weeks) (*n* = 9 bags per unique treatment).

Duration of the experiment	0	2	4	6	8
	Survival (%)				
*C*. *dactylon* with rhizomes	100	100	100	100	100
*C*. *dactylon* without rhizomes	100	100	100	100	100
*H*. *hirta*	100	100	90	20	20

**Fig 1:**
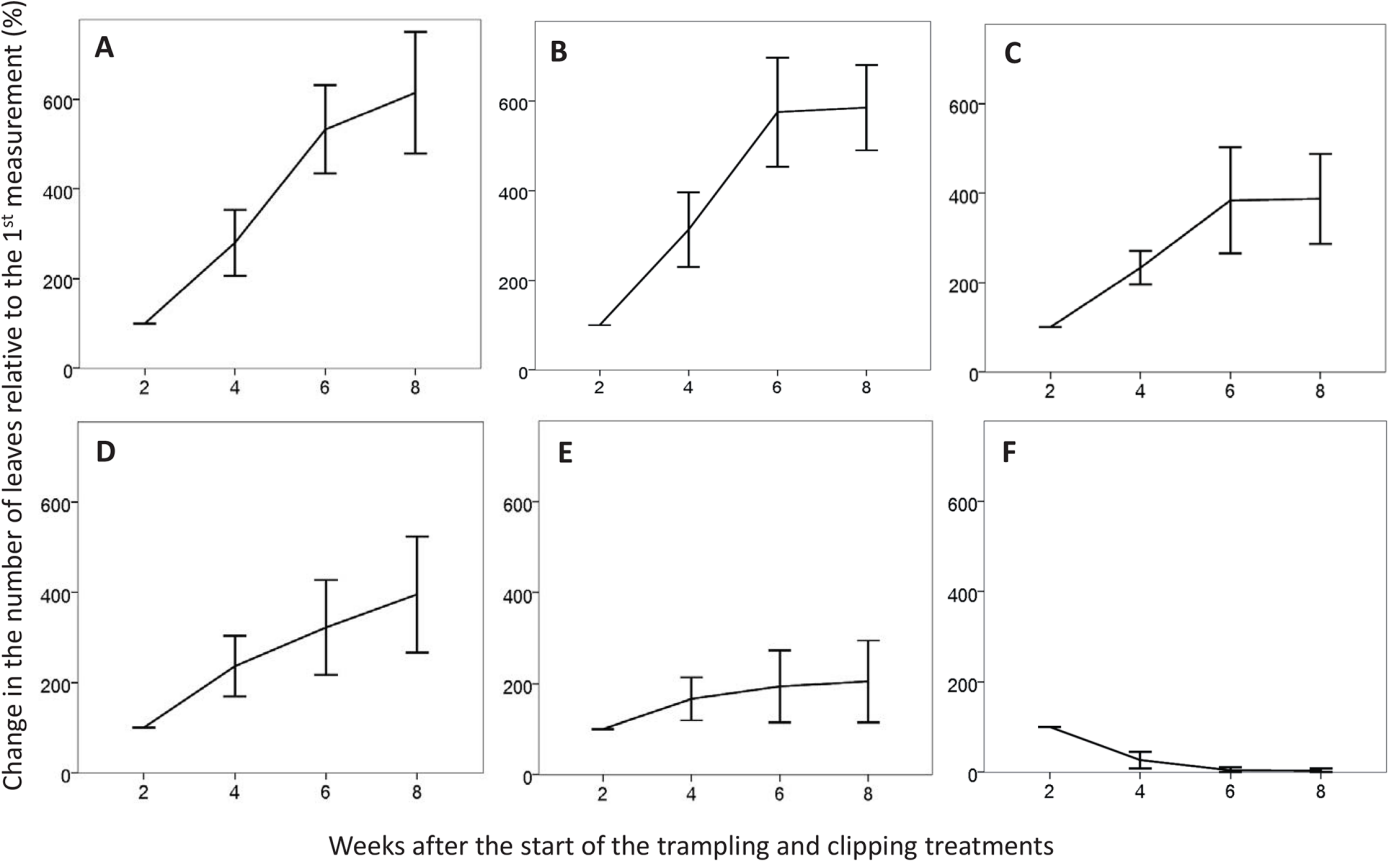
Number of leaves. Proportional change in the mean number (±95% CL) of leaves relative to first measurement (%) over eight weeks’ time. From left to right the graphs show *C*. *dactylon* with rhizomes intact (A and D), *C*. *dactylon* with rhizomes removed (B and E) and *H*. *hirta* (C and F), with the top row displaying the control plants and the bottom row displaying the clipped and trampled plants.

The mean proportional increase of the number of leaves relative to the first measurement showed a clear decline in the number of leaves for the clipped and trampled plants of *H*. *hirta* (Fig 1). Due to the large number of mortalities for *H*. *hirta* (and hence absence of data over the entire period), this species was not taken into consideration when analysing how the treatments influenced the number of leaves, the number of resprouts and the biomass.

For *C*. *dactylon*, the group with rhizomes had a higher number of leaves when being trampled and grazed than when rhizomes were missing (two-way ANCOVA, R^2^_adj_ = 0.54, interaction; *F*_*1*,*103*_ = 7.781, *p* = 0.006; Fig 1). The group without rhizomes and regardless of being treated had a lower number of leaves than when there were rhizomes present (*F*_*1*,*103*_ = 11.527, *p* = 0.001), the number of leaves for trampled and grazed groups regardless of having rhizomes was lower than for the untreated groups (*F*_*1*,*103*_ = 82.224, *p*<0.001) and all plants increased the number of leaves over time (*F*_*1*,*103*_ = 34.447, *p*<0.001).

**Fig 2:**
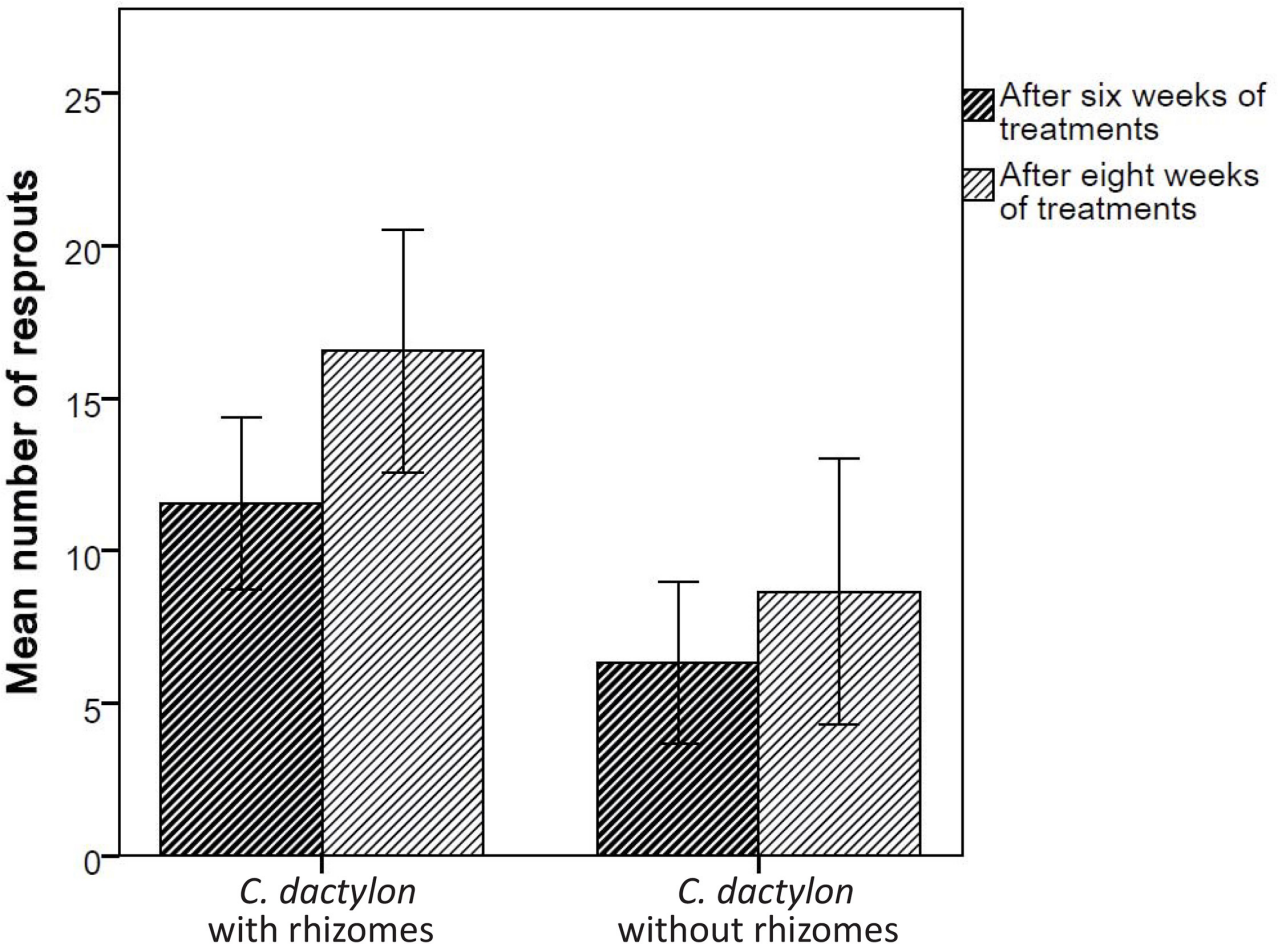
Emerging shoots. Mean number (±95% CL) of emerging shoots or resprouts for *C*. *dactylon* with rhizomes (left) and *C*. *dactylon* without rhizomes (right) after six weeks of treatments (dark hatched bars) and after eight weeks of treatments (light hatched bars).

The number of resprouts was higher for *C*. *dactylon* with rhizomes than for the plants where rhizomes were missing (Poisson regression, Wald Chi-Square = 34.730, *df* = 1, *p*<0.001; Fig 2) and all plants increased the number of resprouts over time (Wald Chi-Square = 11.117, *df* = 1, *p* = 0.001). There was no interaction between the presence of rhizomes and time on the number of resprouts (interaction effect: Wald Chi-Square = 0.045, *df* = 1, *p* = 0.832).

**Fig 3:**
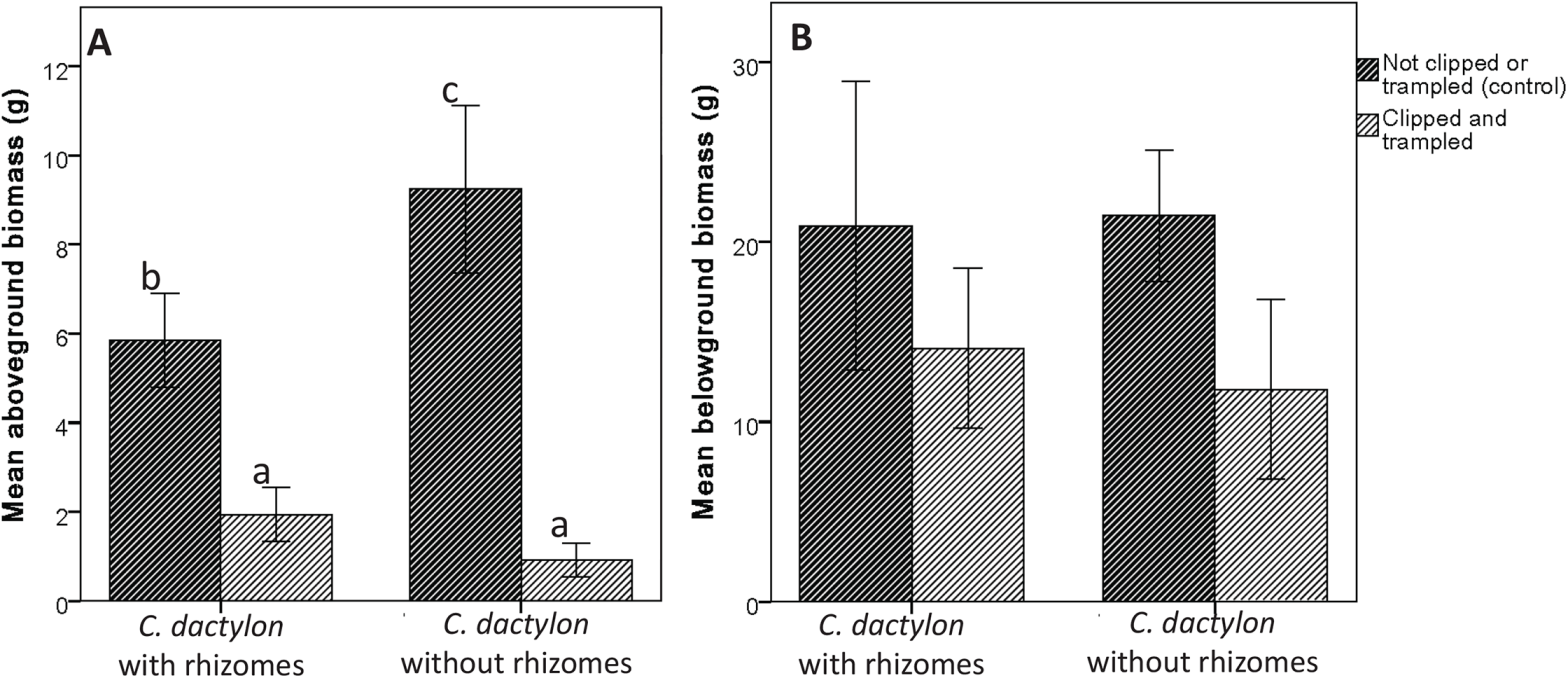
Plant above and belowground biomass. Mean (±95% CL) above (A) and belowground (B) dry weight biomass for *C*. *dactylon* with rhizomes and without rhizomes. Dark hatched bars represent untreated grasses, light hatched bars represent clipped and trampled grasses.

Clipping and trampling reduced the aboveground biomass (two-way ANOVA, R^2^_adj_ = 0.87, *F*_*1*,*32*_ = 205.892, *p*<0.001), but this was larger when rhizomes were missing (interaction; *F*_*1*,*32*_ = 24.179, *p*<0.001; Fig 3A). The presence of rhizomes for *C*. *dactylon* did not have an effect on belowground biomass (two-way ANOVA; *F*_*1*,*33*_ = 0.230, *p* = 0.635; Fig 3B), but was lower for the grasses that received clipping and trampling (R^2^_adj_ = 0.28; *F*_*1*,*34*_ = 14.767, *p* = 0.001). There was no interaction effect of with/without rhizomes x clipping and trampling (*F*_*1*,*32*_ = 1.317, *p* = 0.260).

#### Rhizome Regeneration

**Fig 4:**
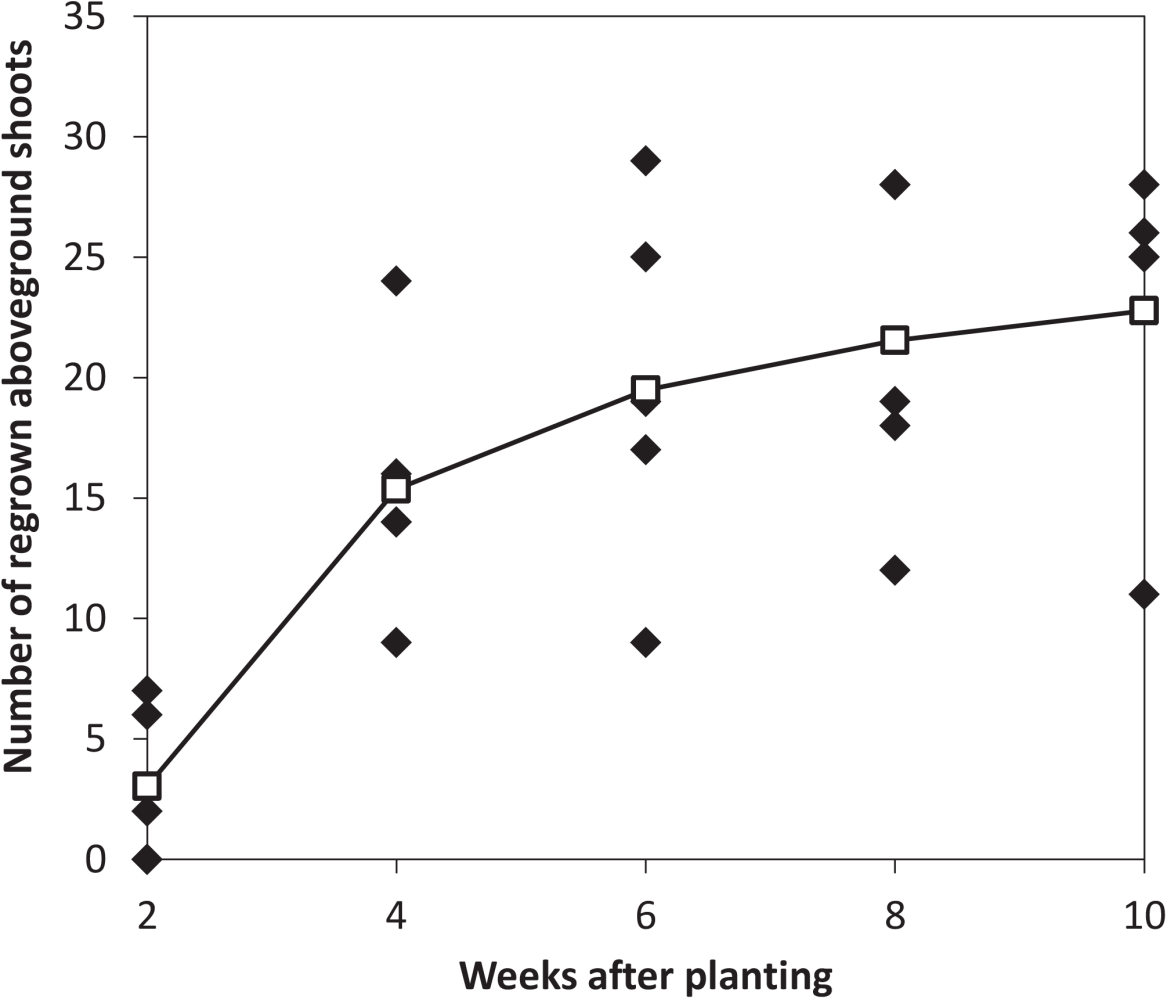
Number of regrown shoots. Observed number of regrown aboveground shoots and best fitted line for each of the five soil bags containing 20 rhizomes. Observations were made every two weeks. The predictions are based on inverse regression.

Over the ten weeks after planting the 100 unconnected, 20 cm long, rhizomes, a steady increase of the number of aboveground shoots was recorded over time (inverse regression; *F*_*1*,*23*_ = 37.790, *p*<0.001; Fig 4; R^2^ = 0.62 against R^2^ = 0.50 for the linear regression), and a large part of the surface of the soil bags became covered with new *C*. *dactylon* shoots. When the experiment was terminated and the rhizomes were taken out of the soil and examined for signs of growth, 52% of all the rhizomes showed at least one developing shoot. Some rhizomes had up to eight growth points.

#### Vegetation Cover, Herbivore Density and Nutrients

The *C*. *dactylon* cover was larger on experimental plots than in the matrix vegetation (respectively 39% and 23% cover; t-test, *t* = 2.422, *df* = 58, *p*<0.02), and mean dung densities were also higher on experimental plots (8.3 and 1.2 dung deposits per sampling point; t-test, *t* = 4.699, *df* = 58, *p*<0.001). However, *H*.*hirta* had a similar cover in experimental plots and in the matrix vegetation (both 12%; *p*>0.05).

**Fig 5:**
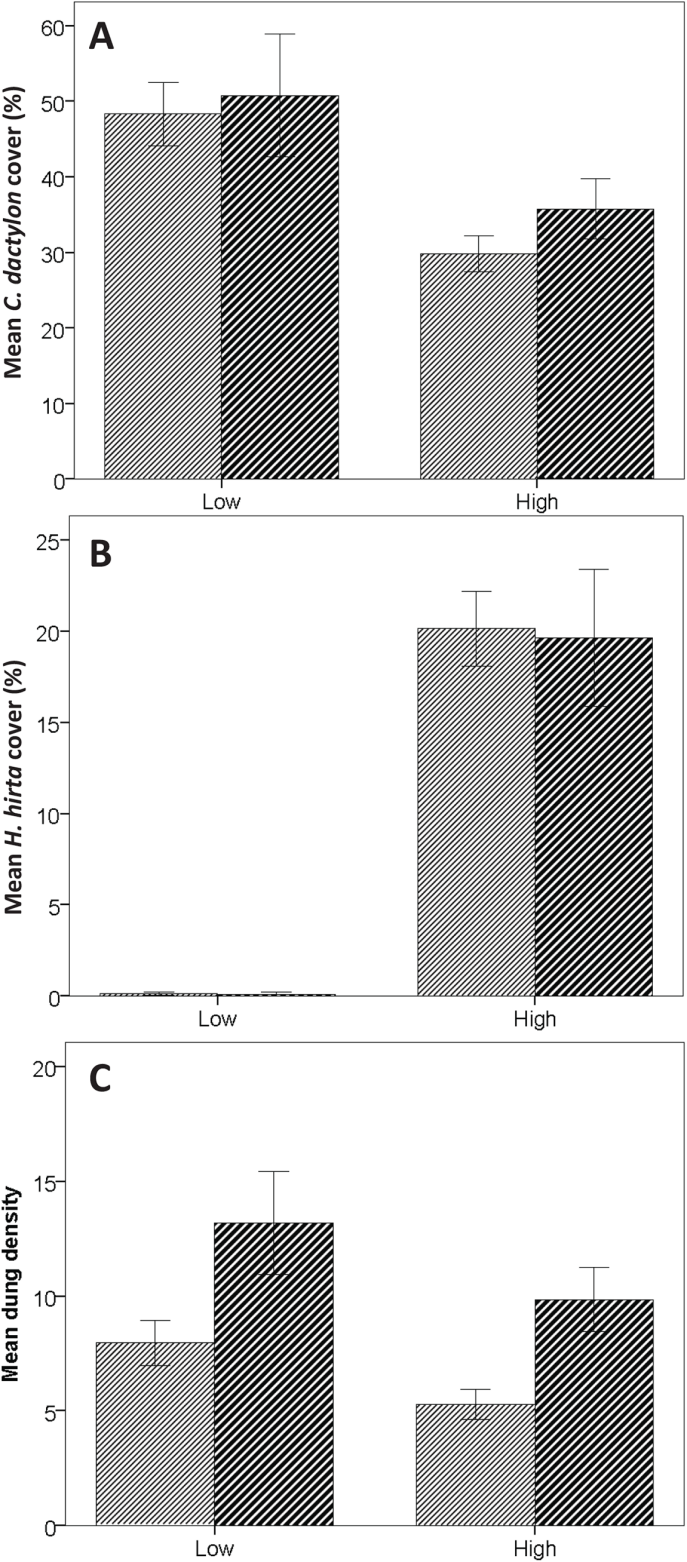
Vegetation cover and dung density on plots. *C*. *dactylon* (±95% CL) (A), *H*. *hirta* cover (±95% CL) (B), and mean dung density (±95% CL) (C) on plots with high and plots with low surrounding vegetation. Dark hatched columns represent central areas and light hatched columns represent edge areas of the 100mx100m plots.

**Fig 6:**
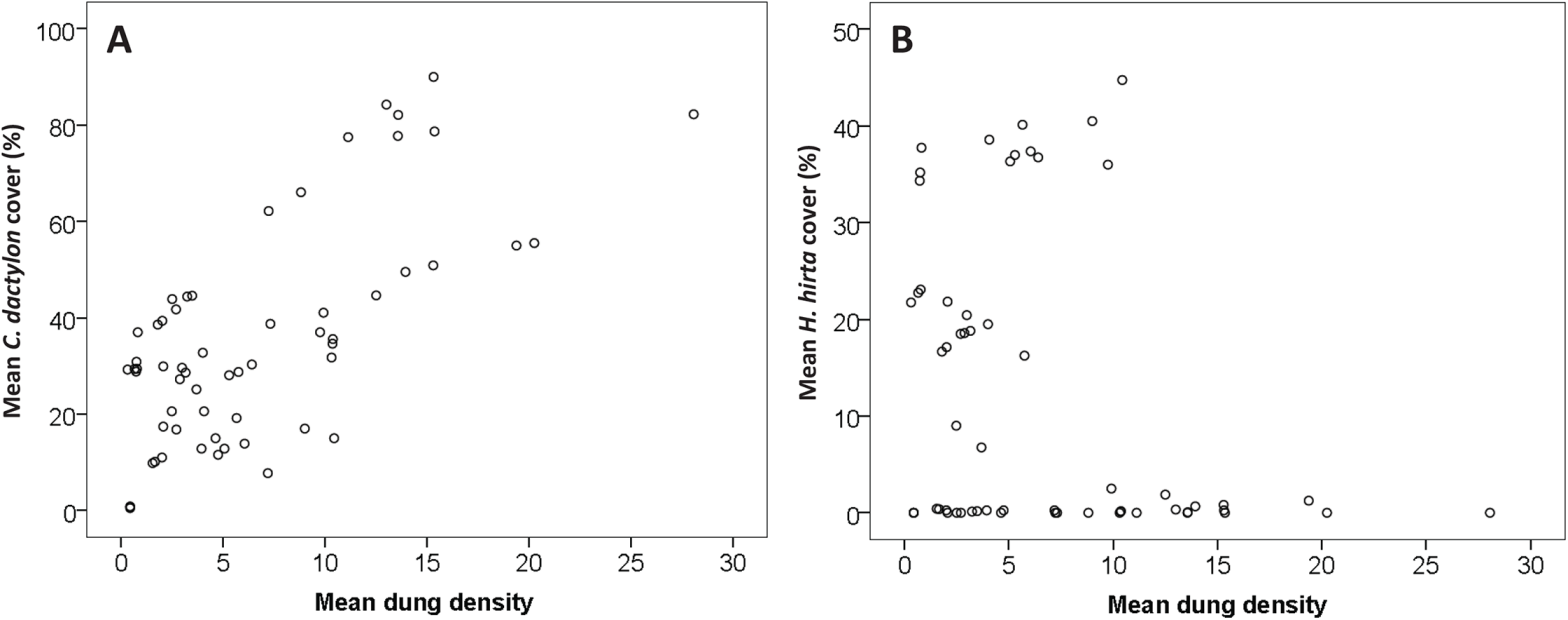
Correlation between vegetation cover and dung density of Zebra and Wildebeest. Relationships between mean dung counts and cover (%) of *C*. *dactylon* (A) and *H*. *hirta* (B).

Among experimental plots, *C*. *dactylon* cover was not affected by centre or edge location, but was higher on plots with low surrounding vegetation (nested GLM, Wald = 54.055, *p*<0.001; Fig 5, S3 Table), slightly higher in plots that were fertilized with nitrogen (Wald Χ^2^_4_ = 9.772, *p* = 0.044), and increased with increasing dung density (Wald Χ^2^_1_ = 154.127, *p*<0.001; Fig 6A).

For *H*. *hirta* also no effect was found of edge or centre location, and also not of fertilization and mowing treatments, but cover increased in plots surrounded by high vegetation compared to plots surrounded by low vegetation (Wald Χ^2^_1_ = 290.301, *p*<0.001; Fig 5), and cover decreased with increasing dung density (Wald Χ^2^_1_ = 11.904, *p* = 0.001; Fig 6B).

ADung densities were not affected by high or low surrounding vegetation, but were higher in plots fertilized with nitrogen (Wald Χ^2^_1_ = 29.128, *p*<0.001), higher in central areas compared to edge areas (Wald Χ^2^_6_ = 57.306, *p*<0.001), and increased with *C*. *dactylon* cover and decreased with *H*. *hirta* cover (Wald Χ^2^_1_ = 77.324, *p* = 0.001; Wald Χ^2^_1_ = 27.455, *p* = 0.001).

For neither grass species and bare soil a significant correlation was found between the available nitrogen and phosphorus concentrations and the cover (Table 4).

**Table 4:** Spearman correlation test results with coefficients (*r*_*s*_) and *p*-values for correlations between soil available nitrogen and phosphorus concentrations and vegetation cover: *H*. *hirta*, *C*. *dactylon*, and bare soil.

	*n*	*H*. *hirta*		*C*. *dactylon*		Bare soil	
Nutrient		*r*_*s*_	*p*	*r*_*s*_	*p*	*r*_*s*_	*p*
Nitrogen	14	0.248	0.394	-0.222	0.446	0.480	0.082
Phosphorus	15	-0.276	0.319	0.246	0.376	-0.367	0.179

## Discussion

Studying the performance of *Cynodon dactylon* (a lawn grass species) and *Hyparrhenia hirta* (a tufted grass species), our results support the hypothesis that lawn formation and its persistence can be induced by sufficient herbivore disturbance. We did not find evidence for the hypothesis that *C*. *dactylon* is favoured by only Nitrogen (N) and/or Phosphorus (P) fertilization. We suggest that the regenerative power of the rhizomes of *C*. *dactylon* underlie this species’ ability to survive under heavy disturbance, i.e. the regenerative ability of *C*. *dactylon* under grazer disturbance enables the species to quickly colonize empty patches of less resilient tufted grasses, facilitating the formation of *Cynodon*-dominated lawns. The tolerance of high grazing levels, and the capacity to regenerate quickly from rhizomes and stolons, probably partly explains why *C*. *dactylon* is such a successful invasive species in other parts of the world [37].

Fertilization treatments did not result in large competitive advantages of either the lawn-forming *C*. *dactylon* or the tufted grass *H*. *hirta*, indicating that competition for nutrients does not directly seem to result in short-term shifts between the two species under the used watering schemes. Small differences in leaf sizes and number of leaves were observed especially for *C*. *dactylon*, but aboveground biomass of *H*. *hirta* did not react to fertilization or competition, and the lower biomass values of *C*. *dactylon* under P fertilization cannot explain the dominance of Cynodon spp. in many grazing lawns [9–11]. Relationships were also absent between the cover of the lawn-forming *C*. *dactylon* or the tufted grass *H*. *hirta*, and the concentrations of available nitrogen and phosphorus in the soil (Table 4), suggesting that nutrients are not the crucial factor in grazing lawn perpetuation. However, water stress could have changed the balance between the two species and interact with nutrients availability and affect species competition [38], and the indirect effect of fertilization through attracting herbivores might be an important indirect factor (see below). However, upon subjecting both species to a combination of heavy trampling and clipping treatments, *C*. *dactylon* displayed a higher resilience than *H*. *hirta* (Fig 1D–1F). The role of rhizomes herein was significant, as the *C*. *dactylon* replicates with their rhizomes intact withstood the trampling and clipping treatments significantly better than the *C*. *dactylon* from which the rhizomes where removed (Fig 1D and 1E). Non-trampled and non-clipped control groups for both of these *C*. *dactylon* groups showed healthy growth, indicating that the removal of rhizomes did not affect the grasses (Fig 1A–1C). The *C*. *dactylon* group without rhizomes withstood the clipping and trampling treatments better than *H*. *hirta*, where there were no rhizomes to make this difference (Fig 1E and 1F). Yet, although nearly all the rhizomes were removed from these experimental plants, there was always a small remainder that could not be removed without disrupting the root system too severely. The removal of the rhizomes should therefore rather be regarded as a vast reduction in the number of rhizomes than as a complete removal, explaining the higher grazing tolerance for the *C*. *dactylon* without rhizomes compared to that of *H*. *hirta*. Fernandez [37] showed that also under field conditions the high regenerative capacity of *C*. *dactylon* rhizomes gives the species a competitive advantage, although this will also depend on the level of disturbance, the prevalence of *C*. *dactylon* in the sward, and the species composition.

We found strong support that the regenerating shoots from rhizomes provide a continuous supply of new green biomass to keep up the photosynthetic machinery, resulting in survival at times of heavy disturbance. *C*. *dactylon* groups with their rhizomes intact had more regrown shoots than *C*. *dactylon* without rhizomes (Fig 2), correlating strongly with the way these different groups withstood the trampling and grazing treatments. The capacity of rhizomes to form resprouts in the regeneration experiment further underlined this finding (Fig 4). Pieces of rhizomes (20 cm) showed a regenerative capacity of 52%, implying that even when all the grasses in a specific area were to lose their aboveground biomass by some major disturbance, more than half of the below-ground residing rhizomes would be able to regenerate at least once.

Since the plots were mown twice a year, the grasses on the plots were shorter than the grasses that surrounded the plots. According to the notion of ‘the landscape of fear’ [32], this influenced herbivore distribution, concentrating herbivores in the central areas of the treatment plots where they seem to experience increased visibility as compared to when they would be residing in the edges of these plots. Herbivore pressure was indeed higher in the central areas of the plots as compared to that in the edges, as shown by the dung distribution (Fig 5B). Furthermore, we found that herbivore density was correlated with *C*. *dactylon* abundance (Fig 6). This is consistent with the hypothesis that *C*. *dactylon* provides herbivores with a higher food intake than when foraging on *H*. *hirta* and also points into the direction of a mechanism where higher herbivore densities, a source of fertilization and disturbance, provide *C*. *dactylon* with a competitive advantage above *H*. *hirta* by suppressing this latter species and thereby reducing competition for *C*. *dactylon*. However, further increasing the grazing level could eliminate the herbaceous layer entirely and/or trigger bush encroachment [39,40].

The studies that showed grazing lawns can be established with mowing treatments [8,19] and/or nitrogen and phosphorus fertilization regimes [12] may have given the impression that mowing or fertilization alone can effectuate such a change due to the specific growth responses of the grasses in question. However, our results show that it is more likely that those treatments merely increased the attractiveness of patches of grass for herbivores, as mowing does by providing fresh regrowth and fertilization does by providing more nutritious leaves. The herbivores that concentrated on specific places, then effectuated the desired changes, being the actual driving force for grazing lawn development, i.e. a source of both nutrients (dung, urine), and disturbance (defoliation through grazing and trampling). It is this combination of fertilization and disturbance under which *C*. *dactylon* is able to outcompete *H*.*hirta*, trigerring the grazing lawn formation. Moreover, nutrients may also exert an effect by enhancing growth rates, which under circumstances favourable to *C*. *dactylon* boost the competitive advantage by accelerating the increasing dominance of the species. Taken together, nutrients may thus serve as both an attractant and a catalyser, which is also confirmed in previous research when fertilized plots showed a more rapid lawn formation [12]. Apart from nutrients and mowing other concentrating mechanisms could be installed such as water points and areas protected from predation [14].

In our study, we demonstrated that *C*. *dactylon* is affected much less by disturbance than *H*. *hirta*. Even though *C*. *dactylon* is not more robust to the direct damage of disturbance, it seems that the regenerative capacity of its rhizomes enables the species to persist after complete removal of its aboveground biomass. Additionally, the effect of nutrients on interspecific competition proved to be small. Even though the effect of disturbance on competition was not studied directly, these conclusions give a strong indication that *C*. *dactylon* benefits from disturbance by reducing competition from another, less resilient species, *H*. *hirta*.

## Supporting Information

S1 Table Soil nutrient concentrations. (xlsx).

S2 Table Common garden experiment dataset. (xlsx).

S3 Table Field survey dataset. (xlsx).
